# Improving Low-Cost Optical PM Sensor Accuracy in Humid Conditions via Aerosol Liquid Water Estimation Using U.S. EPA CSN Data

**DOI:** 10.1021/acsestair.5c00225

**Published:** 2026-01-12

**Authors:** Yuhang Guo, Alexandra Catena, Margaret J. Schwab, Amanda Teora, Oliver V. Rattigan, Violet Harder, Yasaman Hassanzadeh, James J. Schwab, Jie Zhang

**Affiliations:** † Atmospheric Sciences Research Center, University at Albany, State University of New York, Albany, New York 12203, United States; ‡ 101642 New York State Department of Environmental Conservation, Albany, New York 12233, United States

**Keywords:** low-cost optical PM_2_._5_ sensors, mie theory, optical calibration, high RH, aerosol liquid water

## Abstract

High ambient relative humidity (RH) poses a substantial challenge to the accuracy of low-cost optical sensors used for measuring the fine particulate matter (PM_2.5_) concentration. In this study, we developed a novel, practical, and feasible framework for mechanistically correcting low-cost PM_2.5_ sensor measurements under high-humidity conditions by quantitatively separating aerosol liquid water mass (ALW) using the widely available EPA Chemical Speciation Network (CSN) data set, after accounting for the necessary optical calibration procedures that affect sensor performance at elevated RH. We introduced two key correction processes for a low-cost optical PM_2.5_ measurement system comprising a nephelometer and an optical particle counter: (1) optical calibration grounded in Mie theory to account for variations in sensor performance driven by aerosol size distribution, refractive index, and hygroscopic growth, and (2) determination of ALW to estimate dry-equivalent PM_2.5_ mass concentrations under high RH conditions. The corrected PM_2.5_ data exhibit strong agreement with EPA reference measurements, affirming the robustness of the proposed correction framework. Furthermore, the quantification of ALW offers valuable insights for advancing aqueous-phase aerosol chemistry and secondary aerosol formation studies. For regions without colocated CSN data, we provide practical guidance for applying these correction methods using surrogate information. Overall, the methodologies developed in this work are expected to significantly enhance the accuracy and applicability of low-cost optical PM_2.5_ sensors in humid environments.

## Graphical abstract

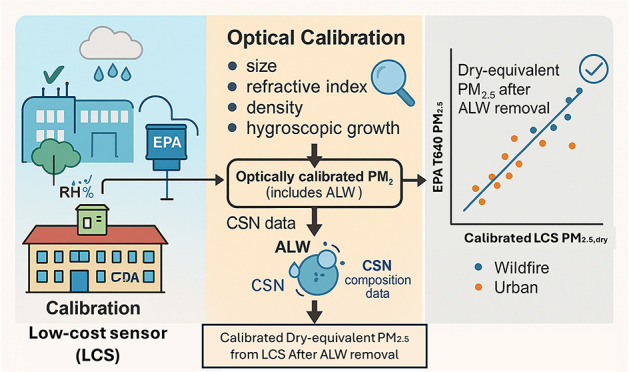

## 1 Introduction

Traditional monitoring of ambient particulate matter with aerodynamic diameters smaller than 2.5 μm (PM_2.5_) primarily depends on certified reference methods established by national and local regulatory agencies, such as the U.S. Environmental Protection Agency (USEPA). These methods provide high measurement accuracy but are often limited in spatial coverage due to high operational costs and logistical deployment challenges.^1–3^ To address these limitations, low-cost optical sensors have been increasingly adopted to complement regulatory monitoring networks. These sensors are cost-effective, compact, and easy to deploy, offering rapid response times and enhanced spatial and temporal resolution. As a result, they are now widely used to assess community-level exposure risks, track wildfire smoke, monitor traffic emissions, evaluate air quality in schools, and assess indoor environments.^4–9^

Currently available low-cost optical sensors can be broadly categorized into two types based on their underlying optical measurement principles: nephelometer-based (Thermo Scientific PDR-1500) or nephelometer-linked sensors (e.g., Plantower PMS5003), which are highly sensitive to PM_1_ (particulate matter with aerodynamic diameters less than 1.0 μm),^10,11^ and optical particle counters (OPCs), such as the Alphasense OPC-N2, which demonstrate greater sensitivity to PM_1_-_2_._5_ (particles between 1.0 and 2.5 μm in aerodynamic diameter).^12,13^ It should be noted that the widely used Plantower PMS5003 was regarded as a nephelometer-like monitor here, given recent findings that its PM_1_ concentration is strongly correlated with the submicron aerosol scattering coefficient, like a nephelometer, while it counted single particle scattering events acting like an imperfect optical particle counter.^11,14^ Nephelometric sensors estimate PM mass concentrations by detecting light scattering intensity at specific angles, typically calibrated using factory-set conversion factors. In contrast, the OPCs detect and size individual particles as they traverse a laser beam, generating particle size distributions that are subsequently converted into mass concentrations. The performance of both sensor types is fundamentally governed by the principles of Mie scattering and is strongly influenced by several particle-specific parameters, including particle size distribution, complex refractive index, the wavelength of the laser source, and the sensor’s scattering angle.^15,16^ These dependencies introduce significant uncertainty, particularly under varying atmospheric conditions, and can considerably limit the accuracy and reliability of the reported mass concentrations.^17–21^

Ambient meteorological conditions, particularly elevated relative humidity (RH), introduce additional uncertainties in the performance of the optical sensors. Unlike regulatory methods, which dry particles to a consistent and low RH, optical-based methods sample and measure particles at ambient RH, which means that the aerosol contains mass from the aerosol liquid water (ALW). High RH promotes the hygroscopic growth of particles, altering their size and refractive index, which in turn affects light scattering behavior governed by Mie theory and compromises sensor accuracy.^22–26^ To mitigate RH-induced bias, empirical correction equations have traditionally been employed,^23,27^ alongside more recent approaches utilizing machine learning (ML) algorithms.^28–31^

Notably, a recent study based on Mie scattering theory has demonstrated that the RH-related bias in nephelometric measurements is quantitatively linked to particle hygroscopic growth^32^ and then ALW content.^24,26^ ALW is strongly dependent on aerosol chemical composition, ambient RH, and temperature, and its mass concentration can exceed two to three times the dry aerosol mass under humid conditions.^24,33^ This dependence highlights the limitations of empirical and ML-based correction models when applied across different regions or seasons, as the ALW mass concentration can vary substantially. To address these challenges, the accurate quantification and removal of ALW from sensor measurements are essential. Separating ALW will not only improve the reliability of PM_2.5_ estimates but also offer critical insights into aqueous-phase aerosol chemistry and secondary aerosol formation processes, including cloud condensation nuclei (CCN) activity.

To separate aerosol liquid water (ALW) from nephelometer-based/like sensors (e.g., Plantower PMS5003, Thermo Scientific PDR-1500), high-time-resolution (minute-to-hourly scale) aerosol chemical composition data are essential. Such data can be obtained from advanced instruments such as the high-resolution time-of-flight aerosol mass spectrometer (HR-ToF-AMS),^24,34^ the aerosol chemical speciation monitor (ACSM),^35^ or the particle-into-liquid sampler (PILS).^36^ However, the high purchase cost of these instruments, combined with the substantial maintenance and personnel requirements for long-term field deployments, makes their use impractical for widespread application within low-cost optical sensor networks.

As an alternative, the U.S. EPA’s Chemical Speciation Network (CSN) provides a widely distributed and long-term record of aerosol chemical composition, albeit at a much lower temporal resolution (typically every 3 or 6 days). In this study, we developed a novel, practical, and feasible framework for mechanistically correcting low-cost PM_2.5_ sensor measurements under high-humidity conditions by quantitatively separating aerosol liquid water mass using the widely available EPA CSN data set, after accounting for the necessary optical calibration procedures that affect sensor performance at elevated RH. The corrected results were validated against the reference-grade EPA monitor to assess the performance of the correction algorithm. In addition, practical recommendations are proposed for extending this framework to regions lacking nearby CSN sites, thereby facilitating broader deployment of low-cost sensors with improved accuracy under humid conditions.

## 2 Methods

### 2.1 Albany Air Quality School Network (AAQSN)

The Albany Air Quality School Network (AAQSN) was established to address the spatial gap left by the limited EPA routine regulatory monitoring stations—currently only one for PM_2.5_ mass concentration and one for ozone concentration, in the New York Capital District region, with the goal to enhance the detection of local air pollutants at the community level. Five K-12 schools were selected as monitoring sites, with each school equipped with two QuantAQ MODULAIR packages—one placed indoors and the other outdoors—to measure indoor and outdoor air pollutants. Two schools are located in the Albany Downtown region, one in the Albany Uptown region, one at Green Island near Troy, and one in Schenectady. AAQSN is designed to address three key objectives: (1) improving the accuracy of low-cost sensors for community-level air pollution measurements, (2) analyzing the spatial distribution of air pollutants at the community level, and (3) investigating the penetration of outdoor air pollutants into indoor environments. This study focuses on the performance of the PM_2.5_ sensors in Objective #1, using outdoor measurements from one Albany Downtown site, which is the closest to the U.S. EPA/New York State Department of Environmental Conservation (NYS DEC) Albany County Health Department PM_2.5_ site (ACHD site) being approximately 140 m (Figure S1), which provided continuous PM_2.5_ measurements using a Teledyne T640 as the reference instrument for calibration in this study, as well as the CSN data set.

The QuantAQ MODULAIR is an integrated, cost-effective sensor package (∼$6000) that incorporates multiple low-cost sensors to measure various air pollutants,^24^ including PM_1_, PM_2.5_, PM_10_, ozone (O_3_), nitric oxide (NO), nitrogen dioxide (NO_2_), and carbon monoxide (CO). For PM_2.5_ mass concentrations, the MODULAIR system integrates readings from a nephelometer (Plantower PMS5003) for PM_1_ and an optical particle counter (OPC, Alphasense OPC-N2) for PM_1−2.5_. To better understand and characterize particle hygroscopic growth under high RH and its impact on MODULAIR PM_2.5_ measurements, two processes were conducted: (1) the optical calibration to correct the performance of PM_2.5_ sensors caused by the factors such as aerosol size, refractive index, density, and particle hygroscopic growth; and (2) separation of aerosol liquid water (ALW) from the optically calibrated data set to derive the dry-equivalent aerosol mass concentration under high RH conditions, as detailed described below.

Besides the PM_1_ and PM_1−2.5_ reading from MODULAIR, the air flow RH and temperature measured by embedded RH-Temperature detectors were also used for the ALW mass concentration calculation, as described in Section 2.2. The time resolution was set to be 1 h, to match the time resolution of EPA/DEC ACHD site measurements.

To avoid analytical bias arising from incomplete observations—particularly the wintertime data gaps in the outdoor QuantAQ record due to solar-panel snow coverage, and ∼6-month availability lag in CSN data, we restricted the analysis to 08/15/2024 to 10/30/2024. This period ensures the overlapping observations between the uninterrupted QuantAQ measurements and 13 CSN sampling days with the key CSN measurement parameters summarized in Table S1. The results presented in this study primarily reflect aerosol characteristics during the late summer and fall seasons. A full two-year analysis will be completed by late 2026, in which we will extend the method developed here to provide a more comprehensive assessment that resolves seasonal variability and incorporates a wider range of source influences.

The Teledyne T640 at ACHD site is routinely operated and quality-assured by the NYS DEC in accordance with U.S. EPA Federal Equivalent Method (FEM) protocols and was used in this study as the reference instrument after constrained by the 24 h Federal Reference Methods (FRMs) data set at the same site (Figure S2). The T640 and the 24 h FRM PM_2.5_ data could be downloaded from (1) “U.S. EPA Air Data: Air Quality Data Collected at Outdoor Monitors Across the US” (https://www.epa.gov/outdoor-air-quality-data) or (2) “Federal Land Manager Environmental Database” (https://views.cira.colostate.edu/fed/QueryWizard/). Based on the fitted relationships, the study period (08/15/2024 to 10/30/2024) was further separated into two distinct regimes: a “wildfire-influenced period” (08/15/2024 to 09/04/2024) and a “local-urban-influenced period” (09/04/2024−10/30/2024). Significant correction was applied for the “wildfire-influenced period” as the T640 substantially overestimated PM_2.5_ mass by nearly 38% during this period, while it matched very well with FRM results during the “local-urban-influenced period”.

### 2.2 CSN Data Analysis and Mass Growth Factor Calculation

The PM_2.5_ component mass concentrations (NO_3_^−^, SO_4_^2−^, NH_4_^+^, and organic carbon (OC)) from the Chemical Speciation Network (CSN, https://www.epa.gov/amtic/chemical-speciation-network-csn) ACHD site were used to determine their dry mass fractions, which were then used to calculate the ALW under high RH conditions, as well as the mass growth factor (MGF). The MGF is defined as the ratio of (1) the total particle mass, including aerosol liquid water, after particle hygroscopic growth to (2) the original measured dry particle mass concentration (also the “dry-equivalent particle mass concentration” in this study). The CSN data could be downloaded from the same website as T640 as well as the 24 h FRM PM_2.5_ data. For this study, an OM/OC ratio of 1.6 was applied to convert CSN OC to organic matter (OM) mass concentration, the same as the value used in the CSN data set for the reconstructed PM_2.5_ mass concentration of the ACHD site. However, the OM/OC ratio can vary substantially depending on emission sources, monitoring location, season, and meteorological conditions, with reported values ranging from approximately 1.4 to above 2. Therefore, using a constant ratio of 1.6 may introduce some uncertainty into our estimates for the MGF, which is further discussed below.

It should be noted that the CSN measurements at the EPA/DEC ACHD site were collected on a 1-in-6-day schedule, providing much lower temporal resolution than the continuous PM_2.5_ measurements from both the MODULAIR unit and the EPA/DEC ACHD monitor (∼1 h resolution). To address the resulting temporal gaps, we assumed that the dry-equivalent particles between two adjacent PM_2.5_ mass−concentration valley edges (marked by the black points in Figure S2) were subject to similar sources and chemical processing and therefore exhibited comparable mass fractions. Accordingly, the mass fractions derived from CSN measurements (Table S1) were extended to the full period between two valley edges to generate an hourly data set, as illustrated in Figure S2. It should be acknowledged that notable variations in aerosol chemical component mass concentrations may occur during the valley-to-valley periods as a result of intrainterval and diurnal variability.^37,38^ In the present study, however, the mass growth factor (MGF) was used for correction, which depended on mass fractions rather than absolute mass concentrations. As mass fractions have been exhibited comparatively smaller temporal variability than mass concentration,^39^ as well as shown in Figure S2, this treatment is expected to partially mitigate the uncertainties arising from short-term compositional fluctuations, with more discussed in Section 3.5.

### 2.3 Mass Growth Factor Calculation

Based on the determined component mass fraction and assuming a normalized PM_2.5_ mass concentration of 1 μg m^−3^ as the basis, the value of the MGF can be equal to the value of the sum of (1) the ALW associated with inorganic components (ALW_inorg_), (2) the ALW associated with organic components (ALW_org_), and (3) the dry particle mass (normalized to 1). Meanwhile, we simply assumed the mass fraction would be the same for PM_1_ and PM_1−2.5_ in this study, which resulted in the same MGF for these two size ranges. For the aerosol under low RH conditions (RH < 60%), MGF would be 1.

ALW_inorg_ was estimated using the thermodynamic equilibrium model ISORROPIA II.^40^ The model utilized (1) the inorganic component concentrations (NO_3_^−^, SO_4_^2−^, NH_4_^+^, Na+, Cl^−^, Ca^2+^, K^+^, Mg^2+^), calculated from the newly determined component mass fraction and the normalized PM_2.5_ mass concentration (1 μg m^−3^), and (2) the relative humidity (RH) and temperature measured inside MODULAIR. Due to the unknown air mass history, the “metastable” mode was adopted, as to better represent the persistence of a fully aqueous phase under ambient conditions and prevent premature crystallization from being artificially imposed. Meanwhile, *M*_ALW,org_ was calculated based on the empirical equation^41^

1 $${\mathrm{ALW}}_{\mathrm{org}}=k_{\mathrm{org}}\times \rho_{\mathrm{water}}\times \frac{M_{\mathrm{org}}}{\rho_{\mathrm{org}}}\times \frac{\mathrm{RH}}{1-\mathrm{RH}}$$

where *k*_org_ is the organic particle hygroscopicity parameter, set to 0.12 as the initial value,^42^ *ρ*_water_ is the water density (1.0 g cm^−3^), *M*_org_ is the organic component concentrations calculated form the organic mass fraction from CSN and the reference PM_2.5_ mass concentration (1 μg m^−3^), *ρ*_org_ is the organic aerosol density (1.4 g cm^−3^),^41^ and RH is the relative humidity reported by MODULAIR. It should be noted that, in this study, a RH threshold of approximately 60% was applied to distinguish between “low RH” and “high RH” conditions. This choice assumes negligible hygroscopic growth below 60%, which is higher than the commonly reported threshold of ∼40% by Li et al. (2021),^43^ who found that the enhancement in aerosol scattering (i.e., f­(RH) > 1) typically begins when RH exceeds ∼40% in urban environments. The 60% threshold was selected as a balance between (1) including more low RH data to improve the robustness of optical calibration (as described in **Step 3** of Section 3.3) and (2) limiting the uncertainty introduced by neglecting ALW under low RH conditions. At RH being 60%,the calculated ALW mass fraction is approximately 20%, which remains within an acceptable uncertainty range for this study. In the following analysis, aerosol under RH < 60% is classified as “dry aerosol” with ALW being 0 and MGF being 1, while aerosol under RH ≥ 60% is referred to as “wet aerosol”.

With knowledge of ALW (= ALW_inorg_ + ALW_org_), the overall ambient particle density after particle hygroscopic growth can be obtained as

2 $$\rho_{\mathrm{ambient}}=\frac{1+\mathrm{ALW}}{\frac{M_{\mathrm{NH}4\mathrm{NO}3}}{\rho_{\mathrm{NH}4\mathrm{NO}3}}+\frac{M_{\left(\mathrm{NH}4\right)2\mathrm{SO}4}}{\rho_{\left(\mathrm{NH}4\right)2\mathrm{SO}4}}+\frac{M_{\mathrm{org}}}{\rho_{\mathrm{org}}}+\frac{\mathrm{ALW}}{\rho_{\mathrm{water}}}}=\frac{\mathrm{MGF}}{\frac{M_{\mathrm{NH}4\mathrm{NO}3}}{\rho_{\mathrm{NH}4\mathrm{NO}3}}+\frac{M_{\left(\mathrm{NH}4\right)2\mathrm{SO}4}}{\rho_{\left(\mathrm{NH}4\right)2\mathrm{SO}4}}+\frac{M_{\mathrm{org}}}{\rho_{\mathrm{org}}}+\frac{\mathrm{MGF}-1}{\rho_{\mathrm{water}}}}$$

where *M*_NH4NO3_, *M*_(NH4)2SO4_, and *M*_org_ are the mass concentrations of particle NH_4_NO_3_, (NH_4_)_2_SO_4_, and organic mass concentration through the mass fraction from CSN and the reference PM_2.5_ mass concentration (1 μg m^−3^), MGF is the calculated following the method as described above based on the normalized PM_2.5_ mass concentration (1 μg m^−3^), the density used for NH_4_NO_3_ is 1.73 g cm^−3^, (NH_4_)_2_SO_4_ is 1.77 g cm^−3^, organic is 1.4 g cm^−3^, and water is 1.0 g cm^−3^. Meanwhile, when RH < 60%, ALW will be 0, and *ρ*_ambient_ will be the dry aerosol density under low RH conditions. In this study, *ρ*_ambient_ is used to represent the aerosol density under both low and high RH conditions, with only the ALW set being 0 at low RH conditions (RH < 60%).

## 3 Results

### 3.1 Overview of Measurements and Correction Processing

Figure 1 illustrates the time series of PM_2.5_ concentrations recorded by the MODULAIR sensor package, alongside hourly PM_2.5_ mass concentrations measured by a T640 instrument constrained by CSN data set at the EPA/DEC ACHD site. A notable discrepancy was observed under high relative humidity (RH) conditions, particularly when RH exceeded 80%. Under high RH conditions (RH ≥ 60%), the PM_2.5_ concentrations measured by the EPA/DEC ACHD site T640 were, on average, only 60% of those reported by MODULAIR—significantly lower than the mean ratio of 82% observed when RH was below 60%. These observations highlight the influence of humidity, especially the hygroscopic growth of particles, on the accuracy of the MODULAIR optical PM monitor.

**Figure 1:**
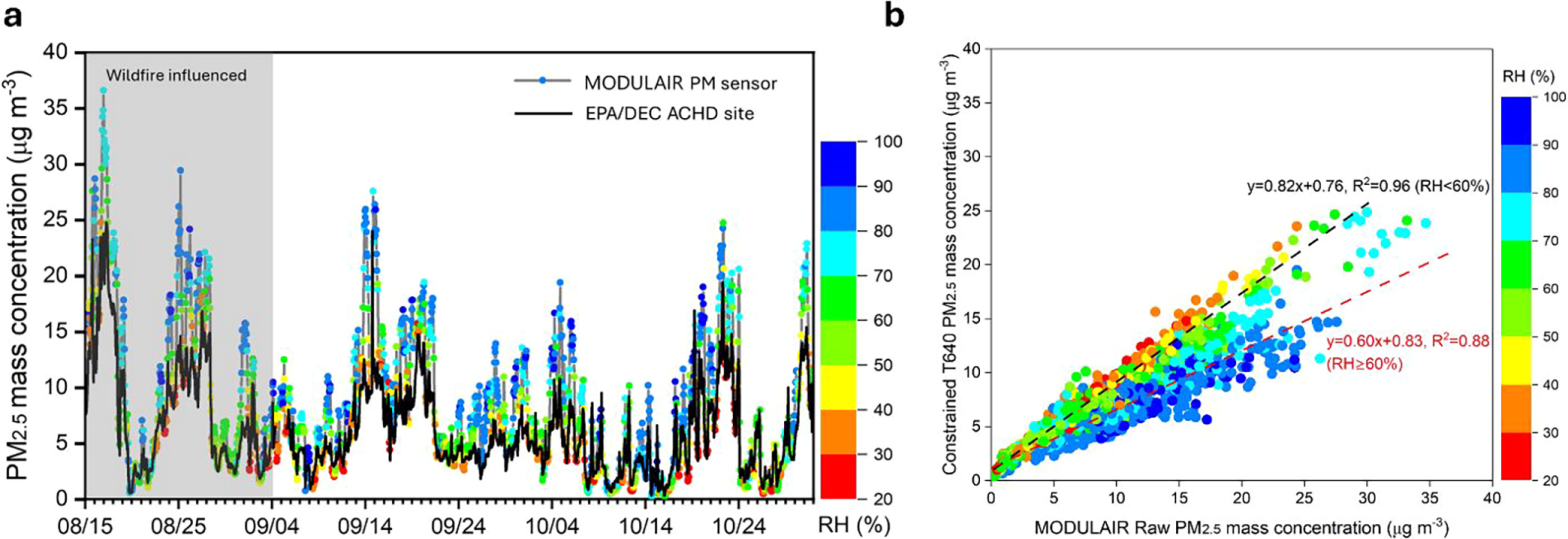
Overview of the measurements as well as their correlation. (1) Time series of PM_2.5_ concentrations reported by MODULAIR, color-coded by RH, alongside PM_2.5_ mass concentrations from the EPA/DEC ACHD T640. (2) Scatter plots illustrating the correlation between MODULAIR-reported PM_2.5_ and EPA/DEC ACHD T640 PM_2.5_ mass concentrations with data points color-coded by RH.

To address the discrepancies under high RH conditions, two key correction procedures were implemented, as summarized in Figure 2. First, optical calibration was applied to MODULAIR data to account for particle optical-related properties such as size distribution, refractive index, density, and hygroscopic growth (**Steps 2** and **3** in Figure 2). Second, the aerosol liquid water (ALW) component was then separated from the optically calibrated values to obtain the dry-equivalent aerosol mass concentration (**Step 4**). The dry-equivalent PM_2.5_ concentration under high RH conditions refers to the particle mass measured after the aerosol stream has been dried—comparable to the method used in EPA/DEC standard PM_2.5_ measurements. These dry-equivalent concentrations were then compared to the EPA/DEC ACHD measurements to assess the effectiveness of the correction procedure. It is important to note that, in this study, optically measured particle mass under high RH includes ALW as part of the total mass, whereas the dry-equivalent concentration represents only the dehydrated (initial dry) fraction.

**Figure 2:**
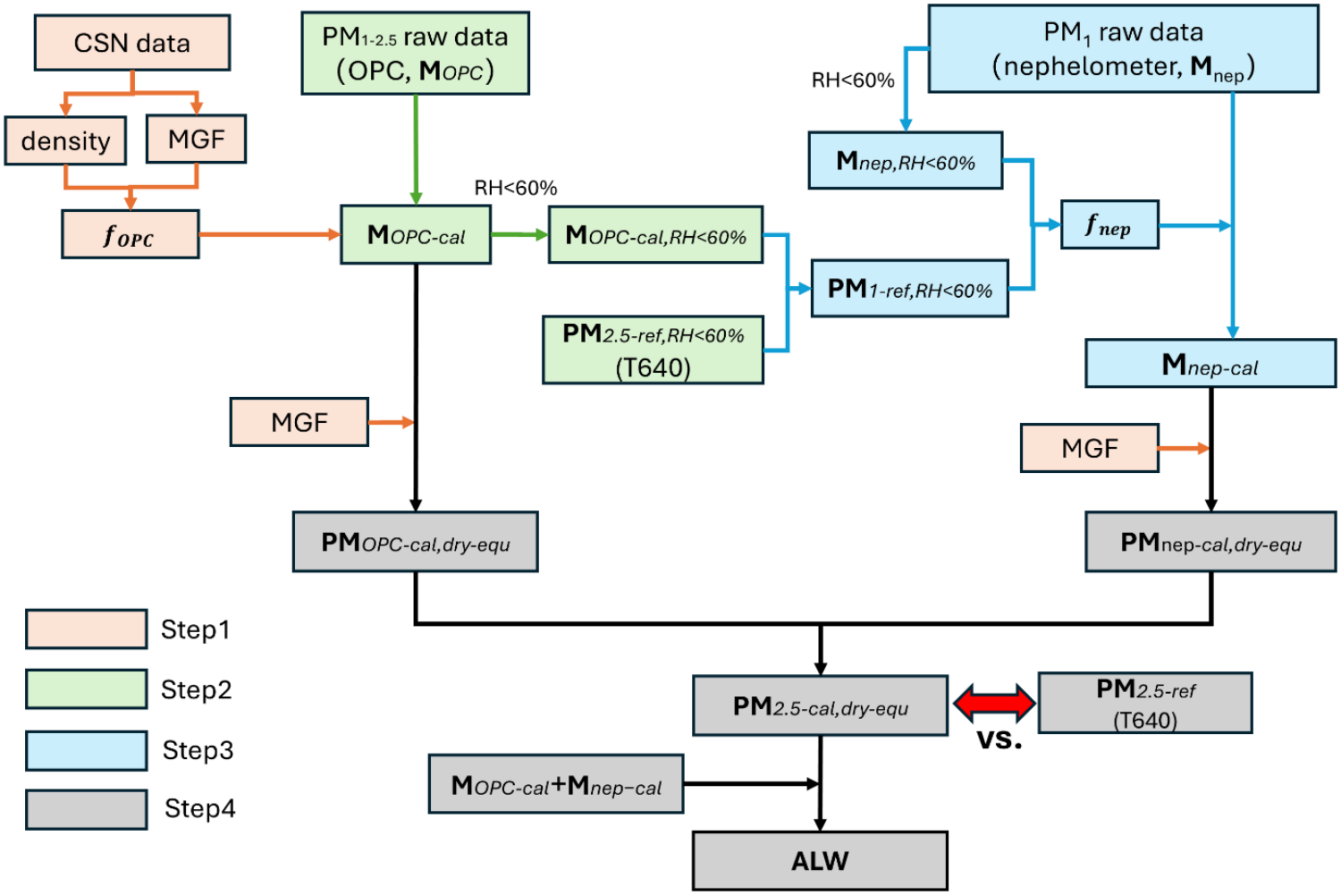
Correction process for the MODULAIR PM_2.5_ data sets (MGF was 1 for aerosol under low RH conditions (RH < 60%)).

### 3.2 Optical Calibration of OPC (“**Step 2**”)

As optical sensors, both nephelometers and OPCs estimate aerosol mass concentrations based on the scattering of light by ambient particles. These sensors are typically calibrated using specific test aerosols—such as monodispersed spheres, under low RH conditions, with defined particle sizes, refractive indices, and densities. As a result, their measurement accuracy under dry ambient conditions depends heavily on the aerosol’s light-scattering properties, which are primarily influenced by particle size and refractive index.

However, under high RH conditions, hygroscopic growth transforms particles from their dry state to a hydrated state by incorporating water. This process increases particle size while reducing their refractive index and density, thereby altering their light-scattering characteristics compared to those under dry conditions. To account for these variations, we define an optical calibration factor $f=\frac{M_{\mathrm{ref}}}{M_{\mathrm{opt}}}$ , where *M*_ref_ is the reference mass concentration from the EPA/DEC measurements in this study and *M*_opt_ is the value reported by the optical sensor. This factor reflects the performance of the sensor under specific environmental conditions.

Due to fundamental differences in the operating principles of the two sensors, we applied separate calibration strategies: (1) Nephelometer-based sensors infer PM_1_ mass concentrations from the intensity of scattered light, and (2) OPCs count the number of particles in predefined size bins and estimate PM_1−2_._5_ mass concentrations by summing across those bins, as described in the QuantAQ’s Technical Notes: “Introduction to the MODULAIR PM” (https://quant-aq.com/technical-notes).

Therefore, we first examined the calibration factor of the OPC, *f*_OPC_, under both low and high RH conditions. The value of *f*_OPC_, determined under dry conditions (RH < 60%, with justification of this threshold provided in Section 2.2), was then used as a basis to derive the calibration factor for the nephelometer, *f*_nep_.

Hagan et al. (2020)^16^ characterized the relationship between a particle’s scattering cross-section and its size—referred to hereafter as the “**scattering-size relationship**”—which is essential for OPC calibration. In the case of the crystalline OPCs, this relationship enables the assignment of particles to appropriate size bins based on their measured scattering cross sections.

Under both low and high RH conditions, the scattering cross-section of an ambient particle is governed by two factors: (1) its physical size and (2) a scattering-size relationship dependent on the particle’s refractive index. Each measured scattering cross-section is matched against the factory-calibrated curve (originally developed using standard monodispersed polystyrene latex spheres) to determine the particle’s size bin and ultimately calculate its mass contribution.

Accordingly, the optical calibration factor, *f*_OPC_, is obtained using eq 3,

3 $$f_{\mathrm{OPC}}=\frac{\rho_{\mathrm{ambien}t}\times V_{\mathrm{ambient}}}{\rho_{\mathrm{OPC}}\times V_{\mathrm{OPC}}}=\frac{\rho_{\mathrm{ambient}}\times D_{\mathrm{ambient}}^{3}}{\rho_{\mathrm{OPC}}\times D_{\mathrm{OPC}}^{3}}$$

where *ρ*_ambient_ is the density of ambient aerosol under low/high RH conditions and was calculated as described in Section 2.2, *D*_ambient_ is the ambient particle diameter, *ρ*_OPC_ is factory used constant particle density for MODULAIR raw data set (1.65 g m^−3^), and *D*_OPC_ is the determined particle diameter through the factory scattering-size relationship and the detected scattering cross-section by ambient aerosol.

To determine the ratio between the ambient aerosol diameter and the diameter inferred by the OPC calibration (*D*_ambient_/*D*_OPC_), it is necessary to evaluate the “**scattering-size relationship**” of ambient aerosols relative to the factory calibration curve. This comparison heavily depends on the refractive index of the aerosol, which affects its light-scattering properties. The average ambient aerosol refractive index (RI) was estimated using a volume-weighted mixing rule: *RI* = ∑_i_ *^n^RI*_*i*_ × VF_*i*_,^44,45^ where *RI* was the average refractive index, *RI*_*i*_ was the refractive index of components (1.52 for (NH_4_)_2_SO_4_, 1.56 for NH_4_NO_3_, 1.33 for water, 1.55 for organic),^45^ and *VF*_*i*_ was the volume fraction, which were determined based on CSN data set. The estimated ambient aerosol refractive index for low RH conditions is around 1.55, which is very near to the factory calibration used for monodispersed polystyrene latex spheres, being around 1.59. This suggests that the scattering behavior of dry ambient aerosols is reasonably well represented by the standard calibration particles.

However, under high RH conditions, hygroscopic growth causes the inclusion of water in the particles, reducing the effective refractive index. In extreme cases (RH > 95%), the RI can decrease to as low as 1.33, equivalent to that of pure water.

Figure 3 illustrates the scattering-size relationships under three representative conditions: (1) Factory calibration particle (*RI* = 1.59), (2) Dry ambient particle (*RI* = 1.55), and (3) Extremely wet particles with near-complete water content (*RI* = 1.33). The differences in the scattering-size relationships among these scenarios, particularly for particles in the 1−2.5 μm diameter range, were not substantial. Although this analysis involves some simplifications, especially given the complex processing algorithms used by the OPCs to infer mass concentration, the introduced uncertainty is likely minimal. This is primarily because the PM_1−2.5_ fraction contributes less than 20% to the total PM_2.5_ mass under typical conditions.^46^

**Figure 3:**
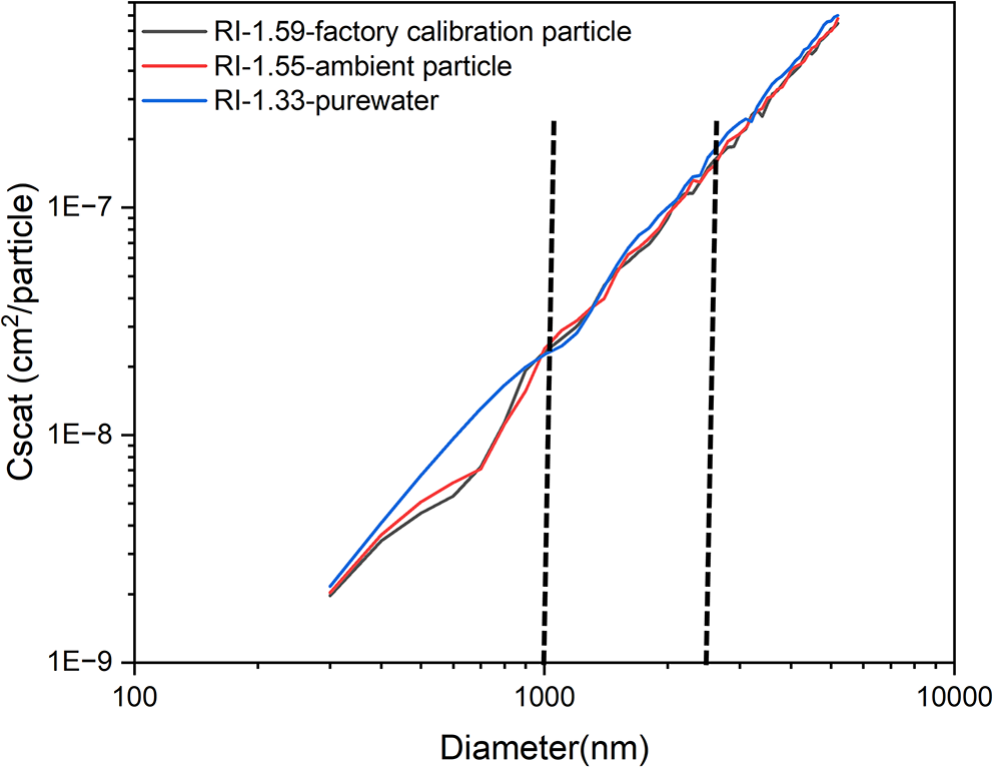
Relationship of the estimated scattering cross-section with the particle diameter (“**scattering-size relationship**”) for different particles.

Therefore, it is reasonable to assume that the inferred particle diameter from the OPC measurements (*D*_OP**C**_) closely approximates the actual ambient aerosol diameter (*D*_ambient_). Under this assumption, the optical calibration factor for OPC, *f*_OPC_, can be simplified as the ratio of the ambient aerosol density to the assumed constant particle density used in MODULAIR’s calculation method (1.65 g cm^−3^), as shown in eq 4

4 $$f_{\mathrm{OPC}}=\frac{\rho_{\mathrm{amebint}}}{\rho_{\mathrm{OPC}}}=\frac{\mathrm{MGF}}{\rho_{\mathrm{OPC}}\times \left(\frac{M_{\mathrm{NH}4\mathrm{NO}3}}{\rho_{\mathrm{NH}4\mathrm{NO}3}}+\frac{M_{\left(\mathrm{NH}4\right)2\mathrm{SO}4}}{\rho_{\left(\mathrm{NH}4\right)2\mathrm{SO}4}}+\frac{M_{\mathrm{org}}}{\rho_{\mathrm{org}}}+\frac{\mathrm{MGF}-1}{\rho_{\mathrm{water}}}\right)}$$

where *M*_NH4NO3_, *M*_(NH4)2SO4_, and *M*_org_ are the mass concentrations of particle NH_4_NO_3_, (NH_4_)_2_SO_4_, and organic mass concentration through the mass fraction from CSN data and scaled using a reference PM_2.5_ mass concentration (1 μg·m^−3^), respectively, MGF is the mass growth factor under high RH conditions and was the sum of the calculated ALW mass concentration based on the reference PM_2.5_ mass concentration and 1 (as described in Section 2.2), and the densities are 1.73 g cm^−3^ for NH_4_NO_3_, 1.77 g cm^−3^ for (NH_4_)_2_SO_4_, 1.4 g cm^−3^ for organic, and 1.0 g cm^−3^ for water. Note that when RH < 60%, ALW will be 0, and *ρ*_ambient_ will be the dry aerosol density under low RH conditions.

Under most circumstances, the calculated ambient aerosol density was lower than the constant particle density used in the factory calibration of the OPC sensor’s factory calibration. The estimated *f*_OPC_ was generally below 0.9 (Figure 4), with a noticeable drop at high RH conditions, reaching values as low as approximately 0.7. Using these estimated *f*_OP**C**_ values, the raw mass concentration reported by the OPC sensor for particles in the 1−2.5 μm diameter range (*M*_OPC_) was corrected to obtain an optically calibrated concentration, *M*_OPC-cal_. This calibrated mass concentration was then combined with the EPA/DEC ACHD PM_2.5_ data set to infer the reference PM_1_ mass concentration (PM_1-ref_), by subtracting *M*_OPC-cal_ from the reference PM_2.5_ mass, and the PM_1-ref_ will be used for the subsequent calibration of the nephelometer measurements, as shown in Figure 2.

**Figure 4:**
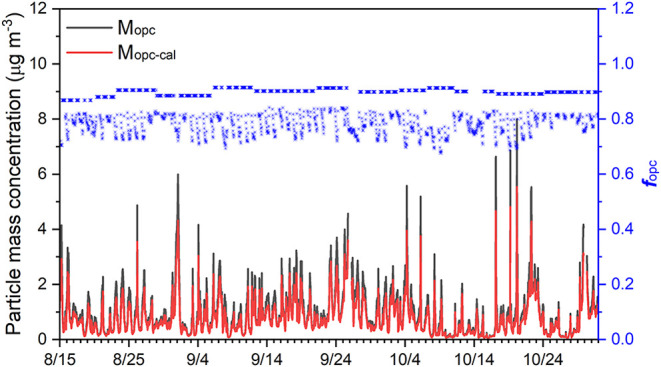
Time series of reported mass concentration from OPC (*M*_OPC_, black line) and the calibrated concentration (*M*_OPC-cal_, red line), along with the estimated *f*_OPC_ (blue points with its corresponding axis on the right).

### 3.3 Optical Calibration of Nephelometer (“**Step 3**”)

Our previous study showed that the total relative scattering intensity detected by the nephelometer for dry particles is proportional to the ratio of values reported by the reference monitor to those from the nephelometer (*f*_nep,RH<60%_).^15^ Assuming a similar aerosol size distribution and refractive index for aerosols originating from similar sources, the *f*_nep,RH<60**%**_ can be obtained through the linear fitting of the measurements from the reference monitor and the nephelometer with similar particle source conditions. Meanwhile, the relative scattering intensity detected by a nephelometer was further verified to be unaffected by aerosol hydration states and exhibited minimal sensitivity to RH fluctuations between 45% and 95%, with an uncertainty less than 5%.^24^

Therefore, the calibration factor derived under low RH conditions can be applied across the entire RH range, as expressed in eq 5

5 $${\mathrm{PM}}_{1-\mathrm{ref},\mathrm{RH}<60\%}=f_{\mathrm{nep}}\times M_{\mathrm{nep},\mathrm{RH}<60\%}+b$$

where PM_1-ref_ can be obtained by subtracting the *M*_OPC-cal_ from the EPA/DEC ACHD site measured PM_2.5_ data set, as mentioned in Section 3.2, *f*_nep_ is the calibration factor for the nephelometer, b denotes the regression intercept, reflecting the intrinsic instrument bias and baseline scattering signal, and accounted for systematic offsets between the nephelometer measurement and the reference monitor, including optical noise and instrument zero drift.

Figure 5 shows the correlations between the PM_1-ref_ values and the nephelometer measurements under low RH conditions (RH < 60%), while the “wildfire-influenced” and “local-urban-influenced” periods exhibit no distinct differences. Therefore, a single *f*_nep_ value of 0.82 and an intercept of 0.71 were obtained by fitting the data with RH < 60% from both periods, and these parameters were applied to all data points within each period to derive the optically calibrated mass, *M*_nep-cal_. Under high RH conditions (RH ≥ 60%), *M*_nep-cal_ represents the total mass concentration of the dry-equivalent particle mass and ALW.

**Figure 5:**
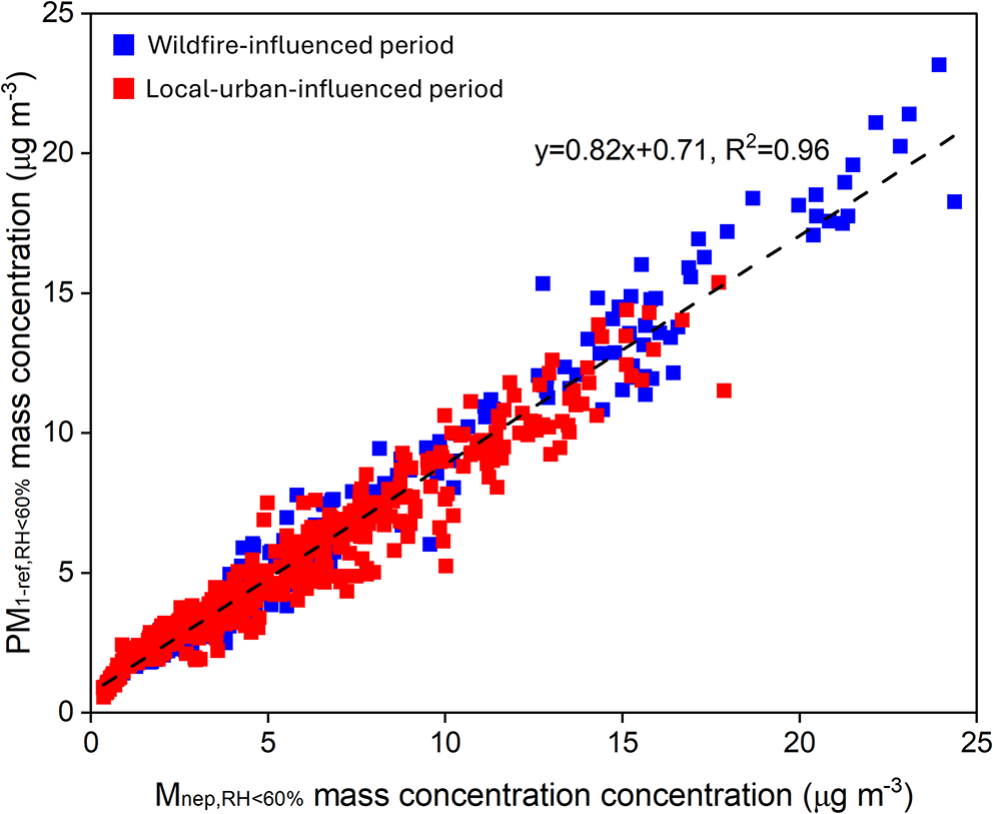
Correlations between the PM_1-ref_ values and the nephelometer measurements under low RH conditions (RH < 60%)

### 3.4 ALW Separation and Method Validation (“**Step 4**”)

With the estimated *M*_OPC-cal_ from Section 3.2 (**Step 2**) and the *M*_nep-cal_ from Section 3.3 (**Step 3**), the derived MGF was then applied to partition the calibrated total mass concentrations from MODULAIR into dry-equivalent particle mass and ALW content. Specifically, (1) the optical calibrated OPC measurement, *M*_opc-cal_, was divided by MGF to yield the dry-equivalent PM_1−2.5_ mass concentration *PM*_OPC-cal,dry-equ_, and (2) the optical calibrated nephelometer measurement, *M*_nep-cal_, was divided by MGF to yield the dry-equivalent PM_1_ mass concentration: *PM*_nep-cal,dry-equ._ The sum of these two dry-equivalent mass concentrations was then defined as *PM*_2.5,dry-equ_, and closely aligned with EPA/DEC T640 PM_2.5_ measurements with a regression slope of 1.02 (Figure 6), validating the method presented in this study. Meanwhile, the difference between (*M*_opc-cal_ *+ M*_nep-ca**l**_) and *PM*_2.5,dry-equ_ was ALW concentration. Notably, the contribution of ALW to the total PM_2.5_ was approximately 30% at RH between 70 and 80%, increased to about 40% at 80−90% RH, and approached or exceeded 50% when RH was above 90% (Figure S4), generally matching the results from previous studies.^47,48^ During the second wildfire plume episode (August 24−27), ALW concentrations exceeded 10 μg m^−3^, underscoring the substantial contribution of ALW under humid and smoke-influenced conditions. These results highlight both the magnitude of ALW occurrence and the significance of separating ALW in this study, providing critical insight for future investigations into aqueous-phase aerosol chemistry,^49^ secondary aerosol formation, and CCN activity.

**Figure 6:**
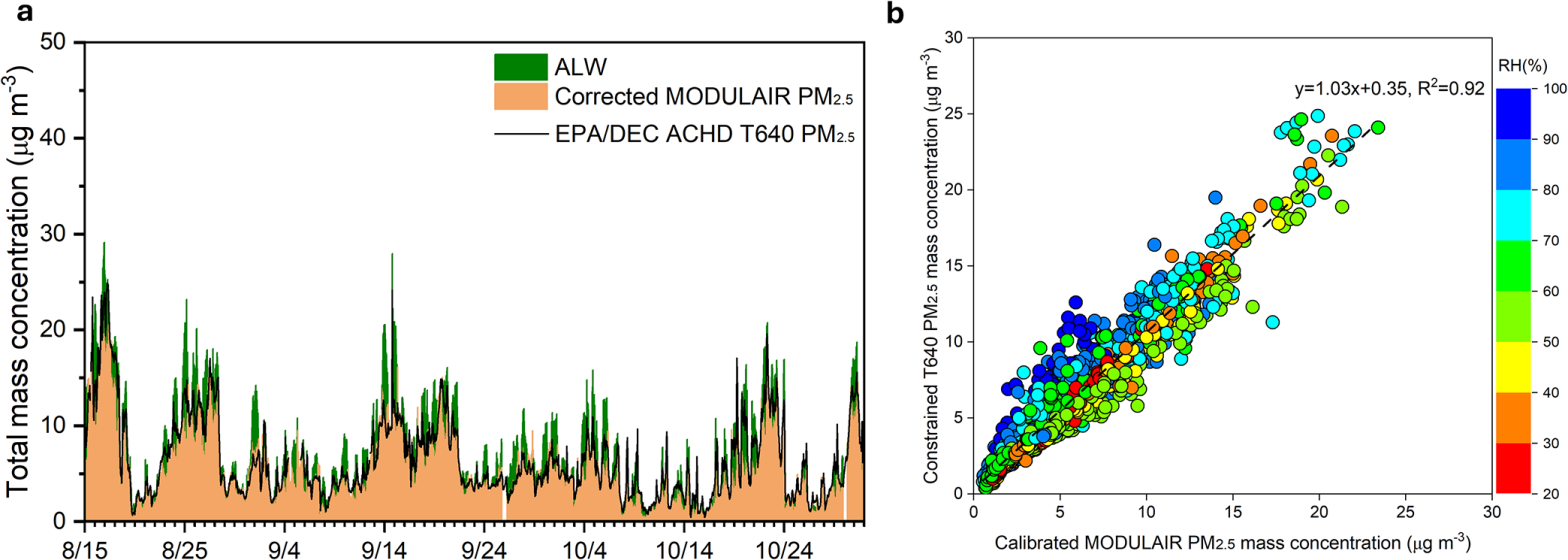
(a) Time series of the separated ALW, the final corrected MODULAIR PM2.5 data, as well as the EPA/DEC ACHD site PM2.5 measurements, and (b) the correlations between PM_2.5,dry-equ_ and EPA/DEC PM_2.5_ measurements being colored by RH.

### 3.5 Uncertainties and Limitations

Among the parameters influencing the MGF calculation, the large variations in (1) OM/OC ratio, (2) the organic hygroscopicity parameter (*k*_org_), and/or (3) the organic aerosol density (*ρ*_org_) represented the major sources of uncertainty. Besides the default value of OM/OC ratio of 1.6, *k*_org_ of 0.12, *ρ*_org_ of 1.4 g cm^−3^, sensitivity tests were performed for (1) the OM/OC with a lower and upper bounds of [1.4 1.8] with default value of *k*_org_ and *ρ*_org_, (2) the *k*_org_ with a lower and upper bounds of [0 0.3] with default value of OM/OC and *ρ*_org_, and (3) the *ρ*_org_ with a lower and upper bounds of [1.0 1.8] with default value of OM/OC and *k*_org_. Sensitivity tests were conducted for each parameter individually. Specifically, [1.4 1.8] was used within the lower and upper bounds of the OM/OC ratio while keeping *k*_org_ and *ρ*_org_ fixed at their default values; *k*_org_ was varied between 0 and 0.3 with default OM/OC and *ρ*_org_; and *ρ*_org_ was varied between 1.0 and 1.8 g cm^−3^ with default OM/OC and *k*_org_. The results are summarized in Table S2, which shows the largest variation of regression slopes between the EPA/DEC T640 PM_2.5_ measurements and the calibrated MODULAIR PM_2.5_ concentrations being approximately 10% when *k*_org_ is 0. This relatively small variation suggests low sensitivity of the calibration outcomes to the tested parameters, supporting the applicability of the default parameter set across different seasons and regions.

In addition, potential volatility artifacts in the CSN measurements—such as NH_4_NO_3_ loss and semivolatile OM evaporation or condensation—could also influence the calculated MGF and consequently the estimated ALW content, leading to additional uncertainty in the estimated *k*_org_. Furthermore, contributions from dust and sea salt were neglected, as these components are expected to be minor for inland PM_2.5_ at this site, though they may be more relevant in other regions.^50^ Finally, the composition mass fraction between weekly CSN samples was interpolated across valley-to-valley periods to represent comparable sources and processing; however, intrainterval and diurnal variability may still occur, and subsegmentation using higher-temporal-resolution proxies is recommended where available. Meanwhile, the simplified assumption of similar mass fractions for PM_1_ and PM_1−2.5_, as well as ignoring the compounds’ mixing state, may also introduce additional uncertainty. However, uncertainty caused by the similar mass fractions for PM_1_ and PM_1−2.5_ was expected to be small, as the relatively dominant mass fraction of PM_1_ to PM_2.5_ (near 80%) for the NY region.^46^ Given that the primary objective of this study was to develop a practical and feasible framework for correcting low-cost PM_2.5_ sensor measurements under high-humidity conditions and obtain a first-order estimation of ALW, the uncertainties associated with the aforementioned assumptions were not explicitly quantified. However, based on the previous sensitivity analysis results, these uncertainties are also expected to remain within an acceptable range. Further investigation using higher-accuracy measurements and advanced instrumentation is warranted to better constrain these effects.

## 4 Discussion

This study developed a novel, practical, and feasible framework for mechanistically correcting low-cost PM_2.5_ sensor measurements under high-humidity conditions by quantitatively separating aerosol liquid water mass (ALW) using the widely available EPA Chemical Speciation Network (CSN) data set, after accounting for the necessary optical calibration procedures that affect sensor performance at elevated RH. It explored two key correction processes for the QuantAQ MODULAIR PM_2.5_ system, which integrates a nephelometer for PM_1_ and an OPC for PM_1−2.5_, including: (1) optical calibration to account for sensor performance variations due to aerosol size, refractive index, density, and hygroscopic growth, and (2) ALW determination from the optically calibrated data set, to derive dry-equivalent aerosol mass concentration under high RH conditions. Following the description of the processes, one of the most critical parameters for the optical sensor correction would be the particle chemical component mass fraction, and it provides essential information for estimating ambient particle density for the OPC optical calibration and deriving the mass growth factor associated with particle hygroscopic growth under high RH conditions, which was then used for ALW separation. Meanwhile, the optical calibration factor for the nephelometer, which is the ratio of reference instrument measurements to the nephelometer, under low RH conditions (“*f*_nep,RH<60%_”), is critical to calibrate the nephelometer for its optical performance under high RH conditions.

Nevertheless, a significant challenge arises from the limited spatial coverage of high-quality reference PM_2.5_ measurements and detailed chemical composition data sets, which constrain the full correction potential of the optical sensors, especially under high RH conditions. Despite this, the key parameters identified—particle size, refractive index, chemical component mass fraction, and *k*_org_—tend to exhibit source-specific consistency. That is, aerosols originating from similar sources generally share stable physical and chemical properties. This source-dependence suggests that large-scale, long-term statistical analyses of aerosol source characteristics can be leveraged to partially overcome geographic data limitations.

Given the widespread use of low-cost sensors such as the Plantower PMS5003 nephelometer and the Alphasense OPC-N2 for particle size distribution measurements, we strongly advocate further research that leverages the sensors which are near regulatory PM_2.5_ monitoring stations and aerosol chemical speciation networks. Long-term colocated data sets offer a valuable opportunity to develop a comprehensive look-up table or reference database—such as the illustrative Table 1 in this study, containing optical calibration factors and correction parameters tailored to specific aerosol source types and seasonal conditions. Such databases would also allow researchers to account for seasonal shifts in aerosol properties due to photochemical aging, as well as being applied to regions without chemical speciation data while with similar source type and geographical characteristics as those represented in the look-up table.

**Table 1:** Summary of the Particle Source and Characterizations for This Study

city	city/rural style, region	particle source	season	*f*_nep_	averaged Chemical fractions (%) (Org/SO_4_/NH_3_/NO_3_/Cl/Na/Ca/K/Mg)	*k*_org_
Albany, NY	Midsized city, Northeastern U.S.	Wildfire	Late summer	0.82	83.9/10.2/1.1/2.8/0.7/0.4/0.3/0.0	0.12
		Local urban	Fall	0.82	72.7/16.4/1.2/6.2/0.9/0.6/0.8/0.5/0.6	0.12

Importantly, our analysis further showed that using the averaged aerosol chemical composition for similar particle source regimes (as summarized in Table 1) introduces only a modest bias (approximately 5%, Figure S5), compared with the ∼3% bias obtained using the valley-to-valley period-averaged method described above. This finding indicates that regime-based average composition is both practically useful and scientifically defensible when local chemical speciation data is unavailable.

In regions without CSN or other speciation measurements, chemical composition can be assigned using a source- and region-informed surrogate CSN site for nonwildfire periods. Specifically, when a target location shares similar urban characteristics, emission profiles, and geographic settings with an existing CSN site, the composition from the most representative site within the same region can be used. For example, Syracuse, NY (located approximately 200 km northwest of Albany) shares comparable urban size and emission characteristics with Albany, making Albany’s CSN composition an appropriate surrogate for nonwildfire conditions. During wildfire events, however, composition was taken from the nearest CSN site, influenced by the same smoke plume, reflecting the regional nature of smoke transport.

Generally speaking, this regime-based approach enables this method to be transferable across regions while preserving the physical consistency of the correction algorithm. Collectively, these advancements are expected to significantly improve the accuracy and reliability of low-cost optical PM sensors under high RH conditions. Moreover, the separation of ALW from particle mass measurements provides additional scientific value by offering insight into aqueous-phase aerosol chemistry including secondary aerosol formation mechanisms and CCN activity.

## Supplementary Material



## Data Availability

The data sets generated and/or analyzed during the current study are not publicly available at this time since they have not yet been fully reviewed by the EPA office, but are available from the corresponding author on reasonable request. The final data will be publicly available at the end of the project.

## Notes

Relative locations of QuantAQ MODULAIR and the EPA/DEC ACHD site (Figure S1), relationship between the ACHD site 24 h FRM PM_2.5_ concentration and the corresponding daily averaged T640 values on 24 h FRM sampling days during “wildfire-influenced period” and “local-urban-influenced period” (Figure S2), valley-to-valley interpolation of CSN-derived mass fractions to generate an hourly data set (Figure S3), time series of the mass fraction of ALW to the total PM_2.5_ (Figure S4), correlations between PM_2.5,dry-equ_ and EPA/DEC PM_2.5_ measurements with PM_2.5,dry-equ_ being calibrated using the averaged aerosol chemical composition for similar particle source regimes (Figure S5), key CSN measurement parameters for the 13 CSN samples used in this study (Table S1), and sensitivity tests results for OM/OC ratio, *k*_*org*_, and ρ_*org*_ (Table S2) (PDF)
