# Supplementary material for: Improving Low-Cost Optical PM Sensor Accuracy in Humid Conditions via Aerosol Liquid Water Estimation Using U.S. EPA CSN Data
Source: ACS EST Air. 2026 Jan 12;3(2):326–35. doi: 10.1021/acsestair.5c00225 (PMC12910586; doi:10.1021/acsestair.5c00225)
Supplement: Supplementary file 1 [file ea5c00225_si_001.pdf]

Supplement for

Improving Low-Cost Optical PM Sensor Accuracy in Humid Conditions via Aerosol Liquid Water Estimation Using U.S. EPA CSN Data

Yuhang Guo[1], Alexandra Catena[1], Margaret J. Schwab[1], Amanda Teora[2], Oliver V. Rattigan[2], Violet Harder[2], Yasaman Hassanzadeh[2], James J. Schwab[1], Jie Zhang[1*]

[1]Atmospheric Sciences Research Center, University at Albany, State University of New York, Albany, NY 12203, USA

[2]New York State Department of Environmental Conservation, NY 12233, USA

[*]To whom correspondence may be addressed. Email: jzhang35@albany.edu

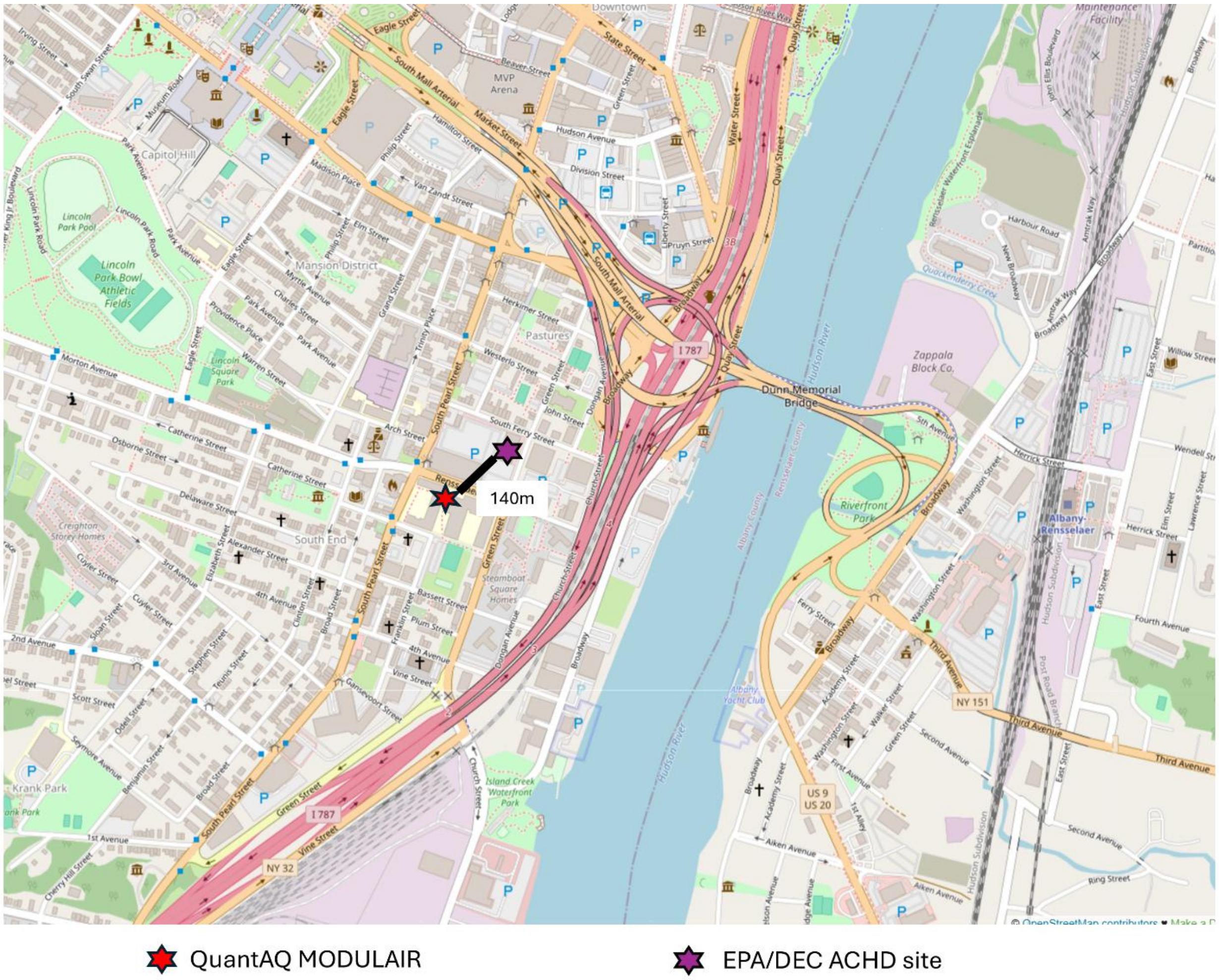

Figure S1. The relative locations of QuantAQ MODULAIR and the EPA/DEC ACHD site. (Map is modified from OpenStreetMap, https://www.openstreetmap.org/, with the free copy right from https://www.openstreetmap.org/copyright)

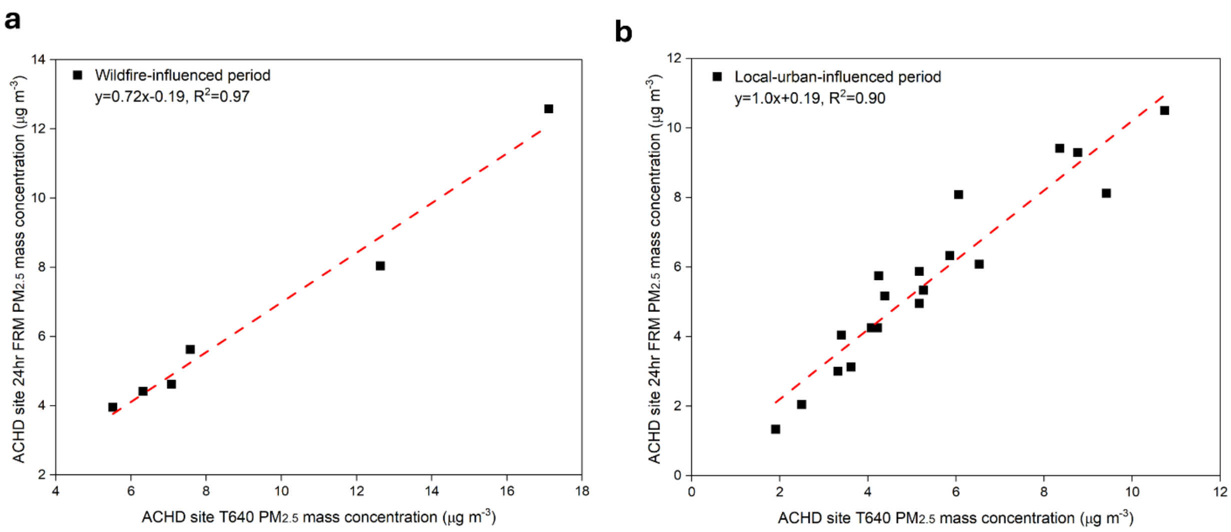

Figure S2. Relationship between the ACHD site 24hr FRM $PM_{2.5}$ concentration and the corresponding daily averaged T640 values on 24hr FRM sampling days during "wildfire-influenced period" and "local-urban-influenced period".

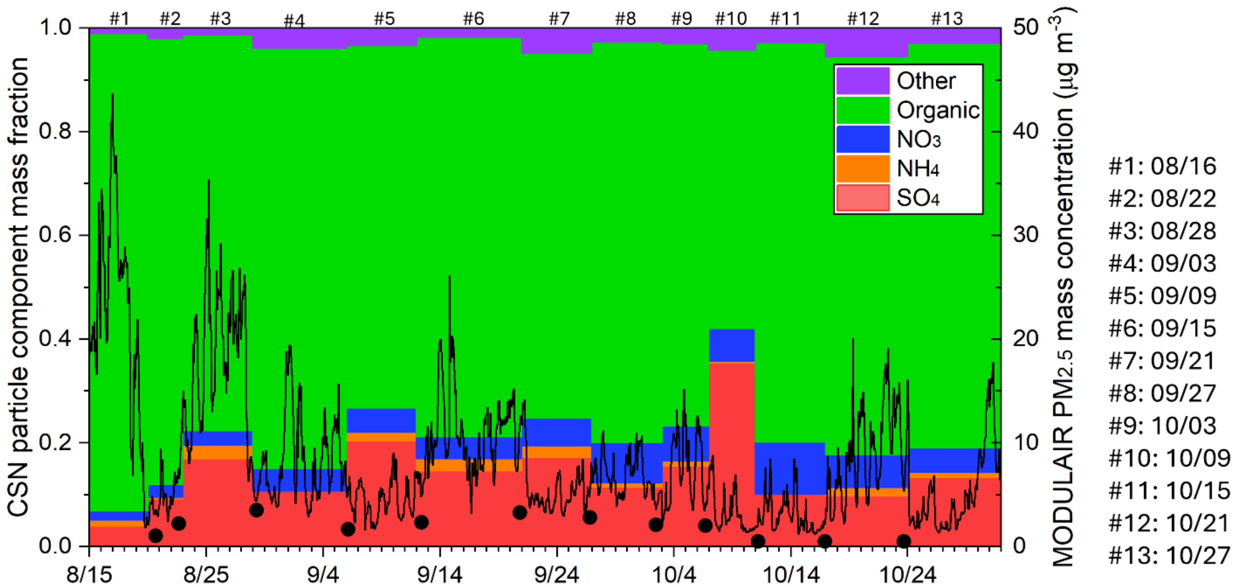

Figure S3. Valley-to-valley interpolation of CSN-derived mass fractions to generate an hourly dataset. The black points indicate the locations of the $PM_{2.5}$ valley edges, and the numbers denote the order of the corresponding CSN sampling days.

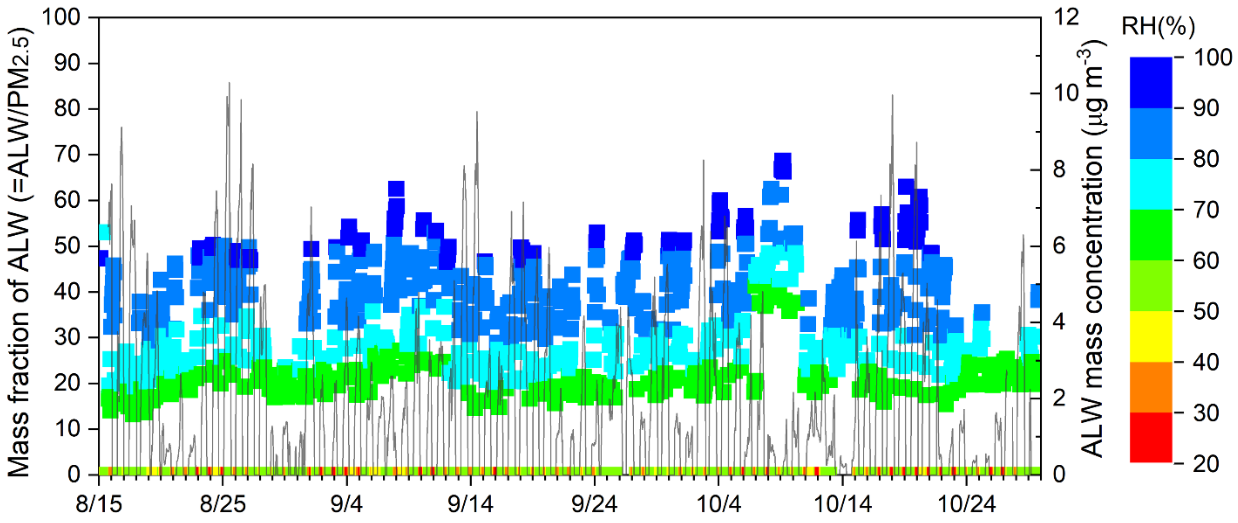

Figure S4. The time series of the mass fraction of ALW to the total $PM_{2.5}$ (=ALW/$PM_{2.5}$), colored by RH, as well as the ALW mass concentration (light grey line)

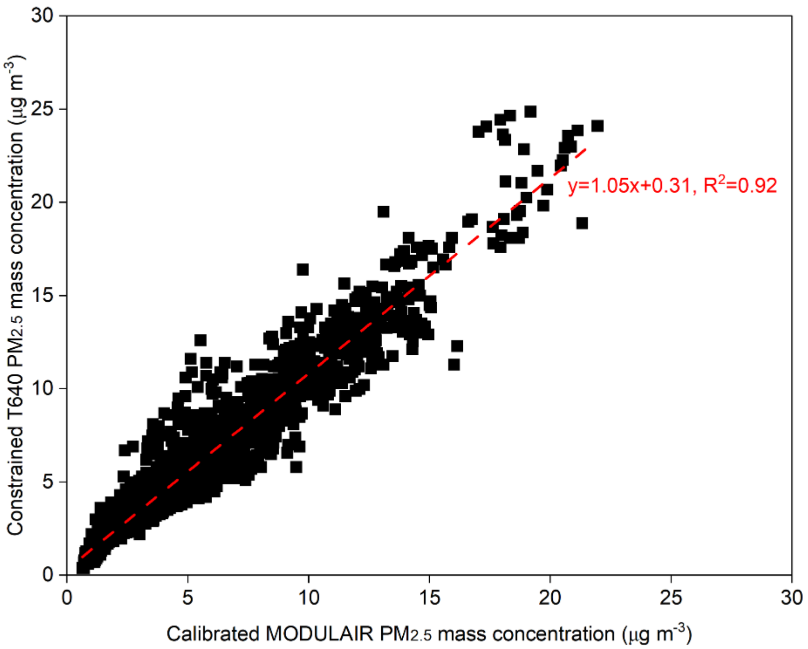

Figure S5. The correlations between $PM_{2.5,dry\text{-}equ}$ and EPA/DEC $PM_{2.5}$ measurements with $PM_{2.5,dry\text{-}equ}$ being calibrated using the averaged aerosol chemical composition for similar particle-source regimes (as summarized in Table 1)

Table S1. The key CSN measurement parameters for the 13 CSN samples used in this study

Date	Na	SO_4	NH_4	NO_3	Cl	Ca	K	Mg	OC	EC	Rec. $PM_{2.5}$
08/16	0.01	0.49	0.15	0.21	0.02	0.06	0.04	0.00	7.12	1.07	13.02
08/22	0.02	0.28	0.01	0.07	0.02	0.02	0.00	0.00	1.62	0.43	3.60
08/28	0.01	0.90	0.14	0.14	0.01	0.03	0.02	0.00	2.51	0.56	5.97
09/03	0.02	0.31	0.00	0.13	0.05	0.02	0.02	0.00	1.47	0.50	3.53
09/09	0.01	0.69	0.05	0.16	0.01	0.04	0.01	0.04	1.48	0.47	4.09
09/15*	0.01	1.11	0.18	0.32	0.03	0.05	0.04	0.00	3.67	0.92	9.70
09/21	0.07	0.75	0.10	0.23	0.08	0.02	0.03	0.00	1.91	0.57	5.12
09/27	0.01	0.47	0.04	0.32	0.02	0.02	0.02	0.04	1.99	0.66	4.90
10/03	0.03	0.47	0.03	0.20	0.02	0.03	0.01	0.00	1.39	0.52	3.92
10/09	0.01	1.10	0.01	0.20	0.04	0.03	0.01	0.03	1.05	0.28	2.99
10/15*	0.00	0.13	0.00	0.12	0.01	0.01	0.00	0.01	0.60	0.21	1.53
10/21*	0.08	0.69	0.11	0.45	0.13	0.10	0.07	0.01	3.37	1.33	8.59
10/27	0.00	0.22	0.02	0.08	0.01	0.00	0.01	0.02	0.82	0.27	1.91

Note: Rec.: Reconstructed

The CSN data for 09/15, 10/15, and 10/21 did not appear in the EPA-released dataset, likely due to unusually high variance during quality-control screening. To minimize data gaps—and because our analysis focuses on mass fractions for the MGF calculation—we used the preliminary datasets provided by NYS DEC for these three dates. These substitutions are considered reasonable, as the retrieved mass fractions are consistent with those on neighboring sampling days (**Figure S3**).

Table S2. The sensitivity tests results for OM/OC ratio, k_{org} and ρ_{org}

Parameters	Value	Fitted relationship	R^2
OM/OC ratio	1.4	y=1.04x+0.39	0.91
	1.8	y=1.01x+0.32	0.93
k_{org}	0	y=0.90x+0.43	0.93
	0.3	y=1.07x+0.84	0.82
ρ_{org}	1.0	y=1.04x+0.38	0.91
	1.8	y=1.01x+0.31	0.93
